# New Veterinary Periodicals in Foreign Lands

**Published:** 1899-06

**Authors:** 


New Veterinary Periodicals in Foreign Lands.—*L'Avenir Vétérinaire*, edited by the retired army veterinarian Chenier. The editor has been well known for a long time in France by his various publications in veterinary journals. He has aimed especially to bring to public notice many abuses which have crept into the army, and has been forced to use many pseudonyms in order not to come under army discipline; but since he has stepped out of active service he wishes openly to bring about a reform of the veterinary service, both military and civil. In his journal only articles which affect the whole service and its governmental relations will be printed. Purely scientific articles are excluded, as there is plenty of room for them in other periodicals. The sheet began with January 1, 1898.

*Revue Médicale Vétérinare et Pharmaceutique de l'Afrique du Nord* likewise appeared January 1, 1898. It appears fortnightly, and aims especially to deal with the diseases, both in man and beast, which occur in the colonies of North Africa and are often carried to Europe. As very little reliable information in regard to these diseases reaches medical Europe, the periodical aims to fill this gap.

*Il Veterinario dei Campagna* is an Italian veterinary review appearing fortnightly, under the editorship of Dr. Cottide-Fabretti, in Comanchio, and Dr. Pietro-Caffaratti, in Villafranca. The journal will not deal with professional matters nor make abstracts from other periodicals, but contain only original articles of a scientific and practical character.

*La Revue des Hongreurs* (Castrator's Review). One could hardly believe that we are *au fin de siècle* when we receive such publications as the one just mentioned, which was founded as the *Cas-*

*trators' Review* in Western France (De-et-Vilaine). It is certainly unique. In the announcement we read as follows: "The organ aims to strengthen the bond of fraternity between the members of the great family called 'Empiricists,' and who are the predecessors of veterinarians." It is due to them, it continues, on account of the many difficulties of their profession, that they receive recognition from society which shall protect them against all attacks from wherever they come. The most noteworthy thing about it is that this organ of cowherds, blacksmiths, quacks, and whatnots should be published by a qualified veterinarian, A. Pinot, in Vitré. He thereby makes himself the advocate and defender of quackery and empiricism.—*Idem.*

